# A Real-Time Reaction Obstacle Avoidance Algorithm for Autonomous Underwater Vehicles in Unknown Environments

**DOI:** 10.3390/s18020438

**Published:** 2018-02-02

**Authors:** Zheping Yan, Jiyun Li, Gengshi Zhang, Yi Wu

**Affiliations:** Marine Assembly and Automatic Technology Institute, College of Automation, Harbin Engineering University, Harbin 150001, China; yanzheping@hrbeu.edu.cn (Z.Y.); zhanggengshi@hrbeu.edu.cn (G.Z.); wuyi_ivy@hrbeu.edu.cn (Y.W.)

**Keywords:** autonomous underwater vehicle, forward looking sonar, obstacle avoidance, reaction obstacle avoidance algorithm

## Abstract

A novel real-time reaction obstacle avoidance algorithm (RRA) is proposed for autonomous underwater vehicles (AUVs) that must adapt to unknown complex terrains, based on forward looking sonar (FLS). To accomplish this algorithm, obstacle avoidance rules are planned, and the RRA processes are split into five steps Introduction only lists 4 so AUVs can rapidly respond to various environment obstacles. The largest polar angle algorithm (LPAA) is designed to change detected obstacle’s irregular outline into a convex polygon, which simplifies the obstacle avoidance process. A solution is designed to solve the trapping problem existing in U-shape obstacle avoidance by an outline memory algorithm. Finally, simulations in three unknown obstacle scenes are carried out to demonstrate the performance of this algorithm, where the obtained obstacle avoidance trajectories are safety, smooth and near-optimal.

## 1. Introduction

Recently, the question of collision avoidance for AUVs has attracted much attention from the ocean engineering and control community due to the wide commercial and military applications of AUVs, such as submarine cable inspection, submarine petroleum pipeline checking, underwater topographic surveying, ocean resource detection and oil field exploitation [1,2,3,4,5]. 

Currently, plenty of approaches were proposed for the obstacle avoidance of AUVs, such as artificial potential field algorithm (APF) [6,7], Dijkstra’s algorithm, A* algorithm, grid method, particle swarm optimization (PSO) [8,9], fuzzy algorithm [10,11,12], neuro-fuzzy algorithm [13,14], bio-inspired algorithm [15], etc. These methods each have their own advantages and special features, while they also have some inherent deficiencies. The artificial potential field method is characterized by a simple structure and easiness to implement, however, it is apt to be trapped in a local minimum when AUVs run into clustered obstacles environments or narrow channels [16,17], the same drawbacks can also be found in genetic algorithm (GA) [18]. Different from the aforementioned approaches, the grid method is especially suitable for terrains with known obstacles due to the capability of searching for the optimal collision avoidance path, however, the storage expense and calculation burden are disadvantages limiting its broad implementation. Motivated by the aforementioned drawbacks in the traditional algorithms, hybrid approaches have been widely presented in the literature. In [19,20], a particle swarm optimization (PSO) with a differentially perturbed velocity hybrid algorithm was proposed for collision avoidance. In [21], a method that combines adaptive fuzzy control and image detection was presented. Other classical methods such as the neural network merging fuzzy inference system can be found in [14].

Most of the above obstacle avoidance algorithms are based on hypothesis that the obstacles are circle-shaped or spherical, however, real obstacles are irregular and in fact they can’t be disposed of simply as circle-shaped or spherical, so those algorithms lack a rapid response to irregular obstacles in unknown situations and are easily trapped in U-shape obstacles. Considering the above problems, a novel real-time reaction obstacle avoidance method is proposed based on FLS. In this strategy, the whole obstacle avoidance process is divided into several steps: directly drive to the target, keep a safe distance, obstacle avoidance, and walk along wall. The algorithm has good flexibility in unknown environments. 

The rest of the paper is organized as follows: Section 2 presents and formulates the obstacle-avoidance problem description. Section 3 presents obstacle avoidance algorithms for different types of obstacles. In Section 4, simulation results are provided to illustrate the performance of the presented algorithm in all sorts of unknown environments. Finally, conclusions are given in Section 5.

## 2. Problem Description and Formulation

### 2.1. Kinematics Model

In this paper, the AUV is equipped with two main propellers mounted astern, which providing navigation power (surge), The AUV is also equipped with four auxiliary thrusters, two auxiliary thrusters are transverse layout providing sway power and yaw momentums, the other two auxiliary thrusters are vertical layout providing power for heave and moments for pitching. In addition, a horizontal rudder and vertical rudder are mounted onboard the AUV to change the heading angle of the AUV in the horizontal and vertical directions, respectively. Accordingly, except for roll, the other five degrees of freedoms such as surge, sway, heave, pitch and yaw are controllable. According to the definition in [22], underactuated AUVs are those where the number of their independent actuators is fewer than their degrees of freedom, so the AUV in the paper belongs to this type.

**Figure 1:**
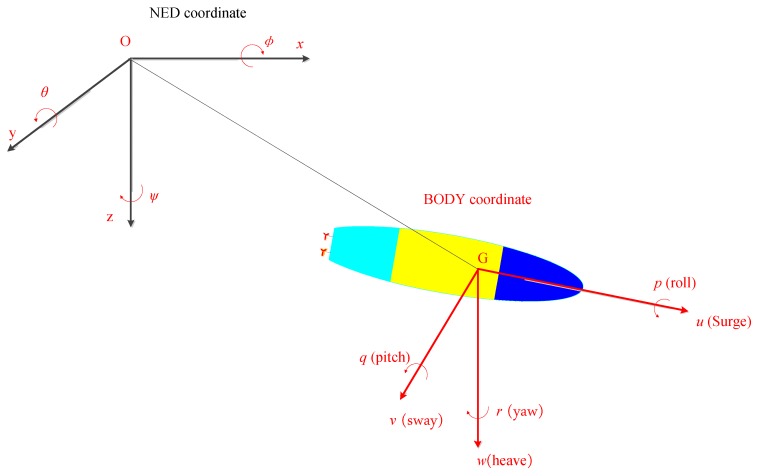
Earth- inertial frames and body-fixed reference frames.

Two reference coordinates are adapted in the paper, they are North-East-Down (NED) coordinate and body-fixed coordinate, as shown in Figure 1, where the linear velocity **V** = [*u*, *v*, *w*]^T^, angular velocity **ω** = [*p*, *q*, *r*]^T^, and attitude **η** = [*φ*, *θ*, *ψ*]^T^.

According to the standard notation motion equations of an AUV, a six degrees of freedom kinematics model for an AUV is described in [23]:

(1) $$\left[\begin{array}{c} \dot{x}\\ \dot{y}\\ \dot{z} \end{array}\right]=J_{1}(\eta )\left[\begin{array}{c} u\\ v\\ w \end{array}\right]$$

(2) $$\left[\begin{array}{c} \dot{\phi }\\ \dot{\theta }\\ \dot{\psi } \end{array}\right]=J_{2}(\eta )\left[\begin{array}{c} p\\ q\\ r \end{array}\right]$$

(3) $$J_{1}(\eta )=\left[\begin{array}{ccc} \cos\psi \cos\theta & \cos\psi \sin\theta \sin\phi -\sin\psi \cos\phi & \cos\psi \sin\theta \cos\phi +\sin\psi \sin\phi \\ \sin\psi \cos\theta & \sin\psi \sin\theta \sin\phi +\cos\psi \cos\phi & \sin\psi \sin\theta \cos\phi -\cos\psi \sin\phi \\ -\sin\theta & \cos\theta \sin\phi & \cos\theta \cos\phi \end{array}\right]$$

(4) $$J_{2}(\eta )=\left[\begin{array}{ccc} 1 & \sin\phi \tan\theta & \cos\phi \tan\theta \\ 0 & \cos\phi & -\sin\phi \\ 0 & \sin\phi /\cos\theta & \cos\phi /\cos\theta \end{array}\right]$$

**Hypothesis** **1:** *Because the roll movement is uncontrollable for AUV, and the structure of AUV is bilateral symmetrical, define* ϕ *= 0, so Equations (1) and (2) can be rewritten as:*

(5) $$\left\{ \begin{array}{l} \dot{x}=u\cos\psi \cos\theta -v\sin\psi +w\cos\psi \sin\theta \\ \dot{y}=u\sin\psi \cos\theta +v\cos\psi +w\sin\psi \sin\theta \\ \dot{z}=-u\sin\theta +w\cos\theta \end{array}\right.$$

(6) $$\left\{ \begin{array}{l} \dot{\theta }=q\\ \dot{\psi }=r/\cos\theta \end{array}\right.$$

In consideration of the fact the AUV’s additional hydrodynamic resistances in the horizontal and vertical direction are greater than those in the longitudinal one, when the speed over grand (SOG) exceeds 1 knot, the propulsive efficiency of the auxiliary thrusters is very low, so the auxiliary thrusters are idle while the AUV is navigating at normal speed. In general, we take *w* = 0, *v* = 0, then Equation (1) is simplified as:

(7) $$\{\begin{array}{l} \dot{x}=u\cos\psi \cos\theta \\ \dot{y}=u\sin\psi \cos\theta \\ \dot{z}=-u\sin\theta \end{array}$$

### 2.2. Obstacle Detection

**Figure 2:**
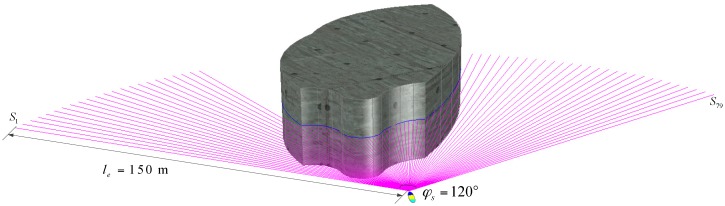
Obstacle detection diagram.

A FLV is utilized for obstacle detection, and the major parameters of the FLS model are designed as follows: the detection range is 150 m; field of view is 120°; detection frequency is 2 Hz; number of beams is 80 beams. As the pitch angle *θ* is seldom altered during normal navigation, the FLS is installed onboard the AUV in the XOY plate. Beams are numbered 0, 1, …, 79 in order, range from (−60°, 60°), and beams’ detection distances are denoted as *S_i_*, *i* = 0, 1, …, 79. The schematic is shown in Figure 2. 

In the paper, a real-time obstacle avoidance strategy is proposed based on FLS, all the obstacles are considered unknown and their shapes are irregular, the obstacles’ outlines are generated by the detection datum of FLS, only the multi-beams in the horizon plane in body coordinates are adopted in consideration of the fact the AUV’s pitch angles are seldom changed. The purple lines are the sonar beams in the horizontal plane in body coordinates, the gray body is an obstacle, and the blue curves are the detected outline of the obstacle. However, the curves, which are also determined by the shape and the detected position of obstacles, are not predetermined even though the environment is known.

### 2.3. Desirable Maximum Turning Radius R_max_

The main specifications of AUV model are as follows: length is 4 m, width is 1.2 m, width is 1.2 m, height is 0.8 m, Max speed is 3.0 m, rated speed is 2.0 m/s. When the AUV navigates at 2 m/s in an underwater environment without ocean currents, the minimum turning radius is about five times the length of the AUV when the rudder angle is set at the largest steering angle of 35°, and it takes about 3.5 s for the rudder angle to vary from 0° to 35°. If the time delay for steering angle transition is taken account of, the trajectory deviation distance is 1–1.5 m, which is small in comparison with the turning radius, to simplify the problem the deviation distance is ignored, in other words, the trajectory of turning is replaced by an arc with a certain radius.

**Figure 3:**
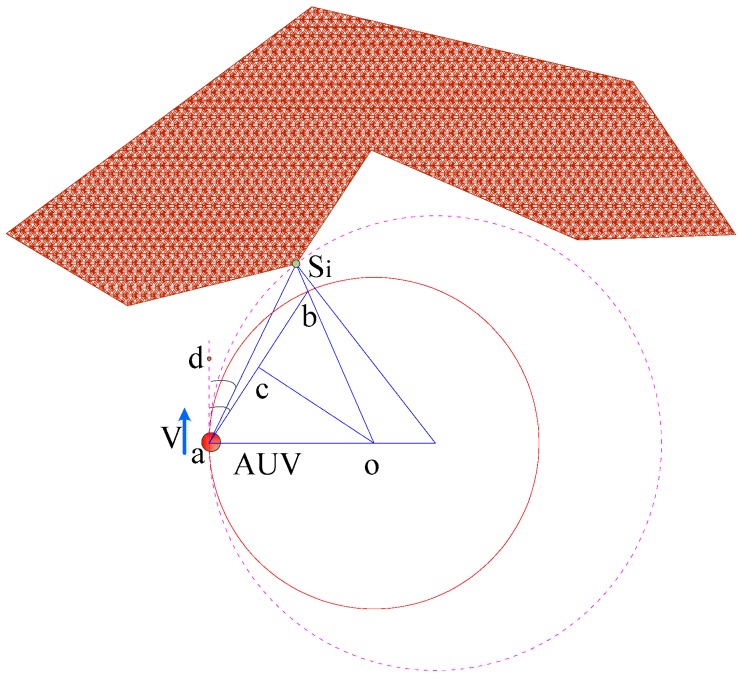
Desirable maximum turning radius for an arbitrary detection point.

In Figure 3, *S_i_* is an arbitrary obstacle point detected by FLS, locating on the right of AUV and the relative bearing angle is α*_i_*, *ρ_i_* is the distance from obstacle point *S_i_* to FLS. *R_i_* is the biggest turning radius for AUV to detour this obstacle point, point O is the center of turning radius, and $\bar{co}$ is the mid-perpendicular of line $\bar{ab}$, *D_s_* is safe distance, and defines:

$$∠daS_{i}=\alpha_{i},\;∠dab=\beta_{i},\;\bar{aS_{i}}=\rho_{i},\;\bar{S_{i}b}=D_{s},\;R_{i}=\bar{oa}.$$

The desirable maximum turning radius *R_i_* is described as:

(8) $$\frac{\bar{aS_{i}}}{\sin(∠S_{i}ba)}=\frac{\bar{S_{i}b}}{\sin(∠S_{i}ab)}$$

(9) $$\left\{ \begin{array}{l} ∠S_{i}ab=\beta_{i}-\alpha_{i}\\ ∠S_{i}ab=\pi -(\pi /2-\beta_{i})=\pi /2+\beta_{i} \end{array}\right.\;$$

Unite Equations (8) and (9), yields:

(10) $$\left\{ \begin{array}{l} \alpha_{i}=\varphi_{s}(i-0.5n+0.5)/n\\ \beta_{i}=\frac{D_{s}}{\rho_{i}}\sec\alpha_{i}+\tan\alpha_{i} \end{array}\right.$$

(11) $$\frac{\bar{aS_{i}}}{\sin(∠S_{i}oa)}=\frac{\bar{oa}}{\sin(∠oS_{i}a)}$$

Substituting Equation (10) into Equation (11), yields:

(12) $$R_{i}=\rho_{i}[\cos\alpha_{i}-\sin\alpha_{i}/\tan(2\beta_{i})]$$

Remark: The AUV postures will change following steering, which makes the hydrodynamic resistance on the AUV increase, and produces roll momentum, and the larger the steering angle is, the larger the effect is. If possible, we choose the steering angle as small as possible.

If the AUV detours around the obstacle from the right side, *i* is the order number of beams, the obstacle point detected by the *i-*th beam whose maximum turning is denoted as *R_i_*, the desirable maximum turning radius1:

(13) $$R_{\max}=\min\{R_{i}|i=40,41,\cdots ,79\}$$

Otherwise, when the AUV detours around the obstacle from the left side, the desirable maximum turning radius is given by:

(14) $$R_{\max}=\min\{R_{i}|i=0,1,\cdots ,39\}$$

### 2.4. Obstacles Classification

**Figure 4:**
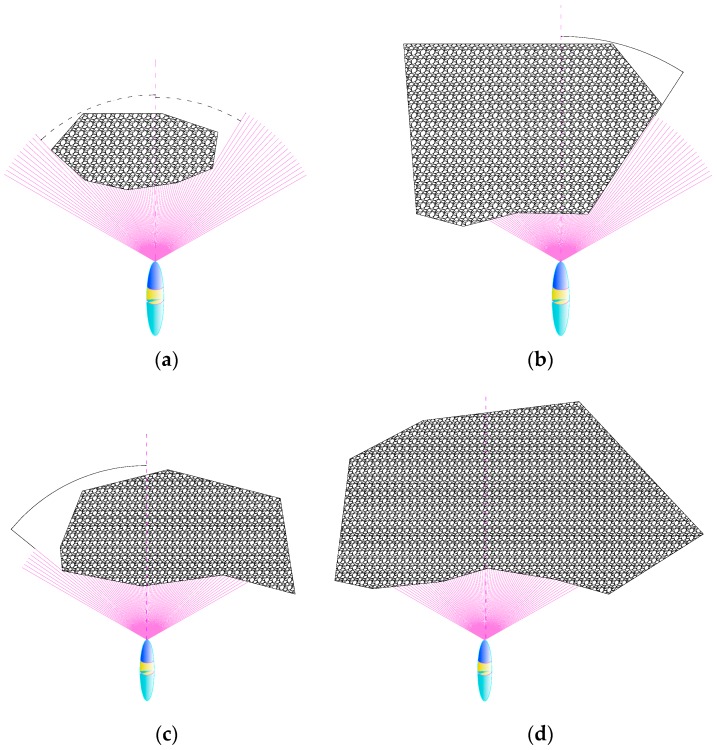
Obstacle classification. (**a**) bounded obstacle; (**b**) left unbounded obstacle; (**c**) right unbounded obstacle; (**d**) unbounded obstacle.

For convenient obstacle avoidance, obstacles are divided into four categories: bounded obstacle, left unbounded obstacle, right unbounded obstacle and unbounded obstacle. the classification criterion is when obstacles enter sonar’s segment areas and their distances to sonar is less than 80 m, according to their visible outline (from the spot of FLS) beyond the detection range of sonar or not, which is illustrated in Figure 4. *k* and *l* is the left boundary and right boundary detected by FLS, respectively, *i*, *j* is serial number of beams, *δ*, *ζ* is arbitrary nature number.

(a) If all the visible outline of obstacle is within the segment areas of FLS, it was defined as a bounded obstacle (BO).
  $$\exists \;k,\;l,\;\delta ,\;\varsigma \in \mathbb{Z},k<l,\;i,\;j\in N,i\in [k,\;l],j\in [k-\varsigma ,\;k-1]\cup [l+1,\;l+\delta ],st.\;0<S_{i}\leq L_{e},\;S_{j}=0$$
(b) If the left edges of obstacle exceed the FLS detection range, and the right ones are within the FLS detection range, it is defined as left unbounded obstacle (LUBO).
  $$\exists \;k,\;\delta \in \mathbb{Z},\;i\in [0,\;k],\;j\in [k,\;k+\delta ],\;st.\;0<S_{i}\leq L_{e},\;S_{j}=0$$
(c) If the right edges of obstacle exceed the FLS detection range, and the left one is within the FLS detection range, it is defined as a right unbounded obstacle (RUBO):
  $$\exists \;k,\;\delta \in \mathbb{Z},\;i\in [0,\;k],\;j\in [k,\;k+\delta ],\;st.\;0<S_{i}\leq L_{e},\;S_{j}=0$$
(d) If two sides of the obstacle exceed the FLS detection range, we define it as a unbounded obstacle (UBO).
  $$\exists \;i\in [0,\;79],\;st.\;0<S_{i}\leq L_{e}$$

### 2.5. Rules of Obstacle Avoidance

**Hypothesis** **2:** The ocean current is below 0.5 knot.

**Hypothesis** **3:** The rudder angle response delay can be ignored.

In general, there are two purposes for steering, one is changing of AUV’s heading angle, the other is counteracting the effect of any external disturbance on the AUV. In this paper, only the first one is taken into account, and collision avoidance is classified into two modes:

Normal obstacle avoidance, which is implemented by steering to change AUV heading angle, regulating the AUV navigation speed appropriately if necessary.

Emergency obstacle avoidance, which is implemented in emergency situations when obstacles are discovered, and part of the obstacle points have entered the smallest safety range of AUV, and it is impossible to avoiding a collision by normal obstacle avoidance; those obstacle points are called emergency collision avoidance points (ECAP) in the paper. The process of emergency obstacle avoidance is composed of the following steps, firstly, the main propellers slow down to zero quickly, then propeller reverse and acceleration, which make the navigation speed of AUV decline rapidly, and auxiliary thrusters will be started once the SOG is below 0.5 m/s. As the emergency obstacle avoidance process is invariable, only normal mode collision avoidance is studied in this paper.

#### 2.5.1. Obstacle Avoidance Rules I

If obstacles are BO, and there isn’t any ECAP when the AUV detours the obstacles, we select the side as detour direction from which the angle deviating from target (ADT) is smaller, the obstacle-avoidance rules described as follows:

(a) If the AUV is currently in line navigation, *γ_l_* is the ADT when detouring around the obstacle from its left side, and γ*_r_* is the ADT when detouring from the other side, and the detour direction is as follows:
  $$\left\{ \begin{array}{l} \gamma_{r}>\gamma_{l},left\\ \gamma_{r}\leq \gamma_{l},right \end{array}\right.$$
(b) If the AUV is currently turning around for obstacle-avoidance, keep the turn direction;
(c) If AUV is currently turning around for reducing ADT, and the turning radius is *R_d_*, the distance to target is *L_v_*, the detour direction is as follows:
  $$\left\{ \begin{array}{l} L_{v}\sin(\gamma_{r})-2\mathrm{sgn}(R_{d})R_{d}{}^{\lambda_{2}}>L_{v}\sin(\gamma_{l}),\;left\\ else,\;right \end{array}\right.$$
  where, sgn(.) is signum function, $\lambda_{2}$ is constant coefficient.

#### 2.5.2. Obstacle Avoidance Rules II

Obstacles are one side or both sides unbounded, and there are no ECAP, so we adopt the following rules:

(a) If the obstacle is LUBO, detour around obstacles from the right side.
(b) If the obstacle is RUBO, detour around obstacles from the left side.
(c) If the obstacle is UBO, and the AUV is turning around, keeping the turn direction, detour around obstacles from the left side; otherwise, detour around obstacles from the right side.

#### 2.5.3. Obstacle Avoidance Rules III

There is ECAP when an AUV detours around obstacles, and we use these obstacle avoidance rules:

(a) If one side of the obstacle is unbounded and exists in ECAP, there is no ECAP on the other side, detour around the other side;
(b) If ECAP only exist on one side of the obstacle and this side is bounded, and the other one is unbounded, select the former as the detour direction;
(c) If ECAP exist on both sides of obstacle, choose the direction to detour from which the ADT is smaller.

**Figure 5:**
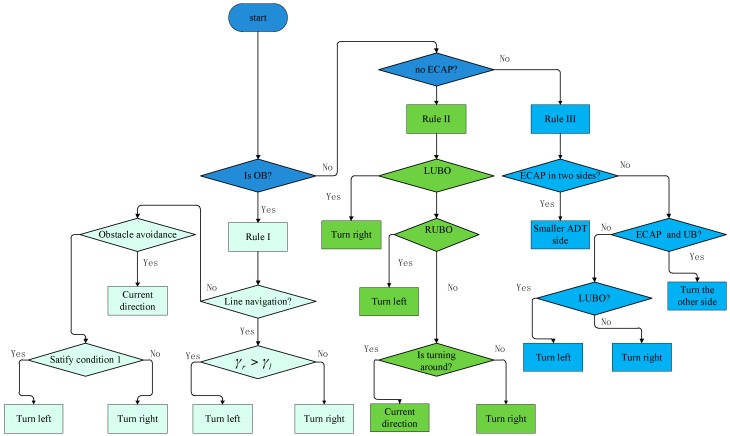
The flowchart for obstacle avoidance rules.

The flowchart for obstacle avoidance rules is shown in Figure 5:

#### 2.5.4. Path Update Principle

If the rudder angle isn’t equal to zero and AUV is detouring obstacles, it is necessary to calculate desirable maximum turning radius *R*_max_ and verify that the current turning radius *R*_c_ is smaller than *R*_max_ for guaranteeing obstacle-avoidance safety, if it is insured that AUV won’t collide with obstacles, keeping the current status, otherwise, a new path needs to be redesigned. If the rudder angle is zero, in other words, the AUV is in straight line navigation, when an obstacle is detected dead ahead of the AUV at less than 80 m (*S_i_* < 80, *i* = 37, 38, …, 42), it is necessary to design a new path immediately, however, the distance is decreased to 60 m (*S_i_* < 40, *i* = 37, 38, …, 42) in dense obstacles environments.

## 3. Obstacle Avoidance Algorithms

### 3.1. Reaction Obstacle Avoidance

RRA is a real-time obstacle-avoidance algorithm, which is especially adapted to unknown complex environments. The process is divided into several steps:

(a) *Directly drive to the target*: when the AUV is passing a certain position, FLS doesn’t detect any obstacles, or detect obstacles which are on the flank of the AUV and the target (or next waypoint) is in the other side, the heading angle is adjusted to the target (or next waypoint) immediately.
(b) *Keep a safe distance*: obstacles are detected on the side of the AUV, so if driving to the target directly, the AUV will collide with obstacles or the safety distance margin isn’t enough, then AUV keeps safe distance from obstacles and decreases the ADT if possible, which makes the AUV drive parallel with the edges of obstacle’s outline.
(c) *Solo obstacle avoidance*: if by keeping the current posture, the AUV will collide with obstacles, then the heading angle must be adjusted to avoid colliding, the detour direction is chosen by the aforementioned rules, reducing the navigation speed if necessary.
(d) *Cluttered obstacle avoidance*: when one of obstacles has entered the 80 m range of AUV in a cluttered environment, this manner is activated, and the AUV selects an appropriate passage between obstacles considering comprehensive factors such as ADT, the width of the passage, and the unobstructed character from the spot of FLS. The AUV keeps a safe distance with the obstacles on the sides of passages, if the passage is wide enough, decreasing the ADT as far as possible.
(e) *Walk along the wall*: if encountering UBO or gallery terrain, the AUV navigates parallel to the edges of the obstacle’s outline until there is no obstacle hampering the AUV from directly driving to the target. During the whole process of walking along wall, some obstacle points need to be saved at intervals as a judgement criterion for terminating this manner. The details will be described in Section 3.4.

### 3.2. Obstacles Outline Disposition

It’s important to choose the right timing for obstacle avoidance. Too early, the obstacles partially enter the detection range of FLS, they aren’t detected entirely; too late, and the AUV gets so close to the obstacle that the obstacle avoidance time is hasty, which creates unnecessary difficulties for collision avoidance. The best time to take obstacle-avoidance measures is when obstacles enter into the 80 m detection range of FLV, except for dense obstacle environments, so the FLS achieves more accurate detection of the obstacles, and the time set aside for collision-avoidance is enough. By this method, not only rudder angle and the time of steering are decreased, but also a more appropriate obstacle avoidance path is produced.

#### 3.2.1. FLS Detection Data Grouping

FLS detection data is saved as an array *β* ∈ **R**^80×3^, where each item of the array denotes the detection distance between sonar beams and obstacle points, and if some item is equal to zero it indicates this sonar beam doesn’t detect any obstacle point. 

**Figure 6:**
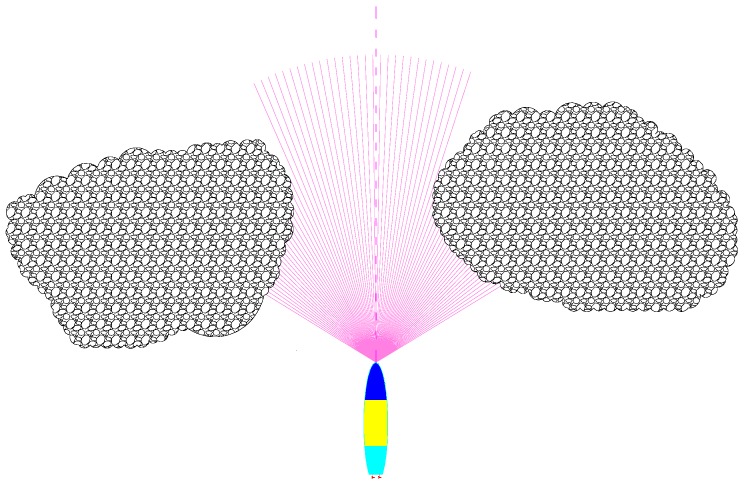
FLS detecting data grouped.

The output datum of FLS needs to be disposed to produce obstacle outline. The first step is taking out the second column date from array *β* and dividing the data into groups according to Equations (15) and (16), and each group data is considered as belonging to an obstacle. The group criterion is as follows:

(15) $$\left\|\bar{S_{i-1}S_{i}}\right\|<d_{b},S_{i}\cdot S_{i-1}\neq 0,i\in [1,79]$$

(16) $$d_{b}=\lambda_{b}l_{e}$$

where $d_{b}$ is gap width, $\lambda_{b}$ is a select coefficient whose value range is [1, 4], $l_{o}$ is the total length of the AUV. All of the points which satisfy Equations (15) and (16) can be considered as a group datapoint of the same obstacle. For example, in Figure 6, the detection data is divided into two groups. 

#### 3.2.2. Largest Polar Angle Algorithm

The output datum of FLS are disperse points in the coordinate system, and an obstacle outline is produced by aligning those points in the same group one by one, however, the outlines aren’t always regular, which can’t be adopted in obstacle avoidance, hence LPAA is proposed to transform the irregular shape into a polygon.

**Figure 7:**
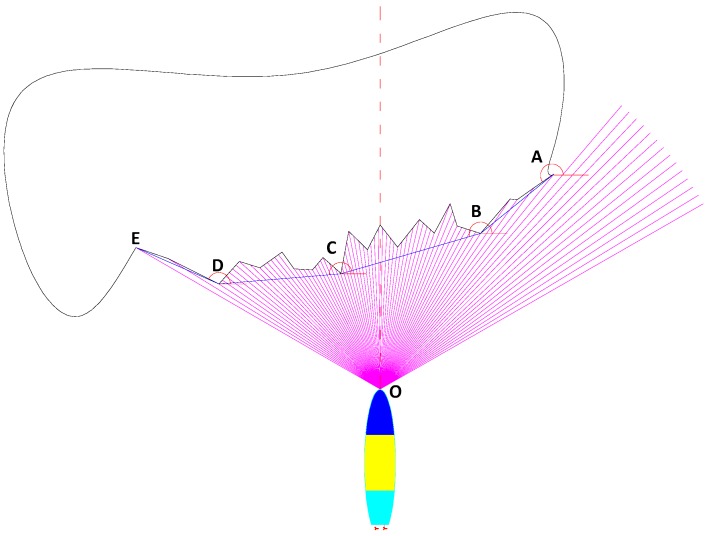
Obstacle detected surface disposed by LPAA.

Figure 7 shows a detected obstacle which is LUBO. In order to improve the efficiency of collision avoidance, it is necessary to simplify the detected outline of the obstacle. The concept of LPAA involves using a convex polygon with fewer sides to surround a group of obstacle points, and satisfy that each vertex of those sides belongs to the array *β*, then the irregular obstacle visible outline is changed into a convex polygon. The polar angle of those sides is decreased gradually according to generated sequence, the specific steps are as follows:

Step 1: Take grouped data to generate sonar beams (purple line) and generate obstacle points;
Step 2: Line up obstacle points one by one to produce a detected obstacle outline (black line), choose the rightmost border point (point A) as the starting point, connect it with others points on the left of it;
Step 3: Find an obstacle point as point B, which satisfies the condition that the polar angle of line AB is larger than those of other points aligned with point A. The polar angle value range is [0°, 360°), which is taken as positive in the anti-clockwise sense without loss of generality;
Step 4: Take the point found in step 3 as the new starting point, repeat step 1 and step 2 to find the next point (point C, D, …) until the leftmost border point is picked out;
Step 5: Align points A, B, … in sequence. The blue line ABCDE is the disposed result.

In practice, once the detour direction is determined, we only need to deal with the points in this direction. Taking Figure 7 as an example, we only need to deal with the obstacle points from *S*_40_ to the rightmost border point when detouring around the right side of the obstacle, which increases the obstacle avoidance response speed by reducing unnecessary computation.

### 3.3. Path Design for Single Obstacle

The aim of path design is achieving the shortest and smoothest paths free of collisions using fewer steering corrections and smaller steering rudder angles if possible. We design several waypoints and connect them by a straight or smooth arc (its radius is the turning radius) to construct a safe path.

**Figure 8:**
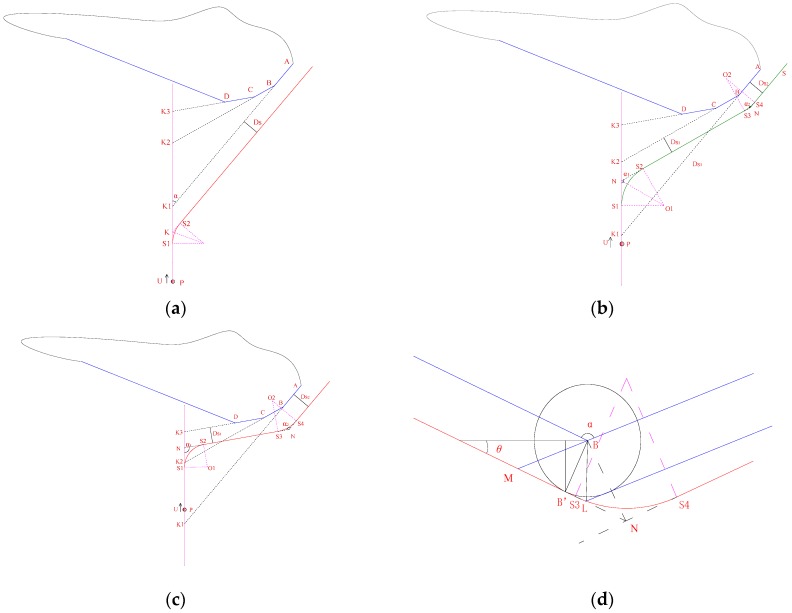
(**a**–**c**) Path design for single obstacle; (**d**) Local amplification diagram.

In Figure 8 the obstacle corresponds to LUBO, and the AUV detours around the right side of the obstacle according to the aforementioned obstacle avoidance rules. Extend the line ab, bc, cd, …, to intersect AUV track line at point *k*_1_, *k*_2_, *k*_3_, …, respectively, and the steps are as follows:

Step 1: Find out the intersection k_1_ between line AB and AUV track line, calculating the length of line segment *Pk*_1_.
Step 2: Calculate $f_{1}(\tau )$:
  (17) $$f_{1}(\tau )=\bar{PK_{1}}-U\cdot T_{\tau }-R_{\min}\cdot \tan(0.5\alpha )-D_{s}\cdot \csc\alpha$$
  where, $T_{\tau }$ is the response time of the obstacle-avoidance system, *U* is the speed of AUV, $R_{\min}$ is the smallest turning radius of AUV, $D_{s}$ is safe distance, take $D_{s}\geq 4L_{o}$
Step 3: If $f_{1}(\tau )$ is negative, turn to step 8, otherwise, calculate $f_{2}(\tau )$:
  (18) $$f_{2}(\tau )=\bar{PK_{1}}-U\cdot T_{\tau }-R_{\max}\cdot \tan(0.5\alpha )-D_{s}\cdot \csc\alpha$$
  where, $R_{\max}$ is the largest turning radius for AUV.
Step 4: If $f_{2}(\tau )$ is non-negative, parallel translation *AK*_1_, and intersect AUV track line at point *K*, $KK_{1}=D_{s}\csc\alpha$, design a transition point *S*_1_ as beginning position of steering, which satisfies:
  (19) $$PS_{1}=PK_{1}-R_{\max}\cdot \tan(0.5\alpha )-D_{s}\cdot \csc\alpha$$
Step 5: If $f_{2}(\tau )$ is negative, the turning radius *R* and transition point *S*_1_ satisfies:
  (20) $$\left\{ \begin{array}{l} R=(PK_{1}-U\cdot T_{\tau }-D_{s}\cdot \csc\alpha )/\tan(0.5\alpha )\\ PS_{1}=PK_{1}-R\cdot \tan(0.5\alpha )-D_{s}\cdot \csc\alpha \end{array}\right.$$
Step 6: Design a transition point *S*_2_ as the steering end position, where the heading angle of the AUV satisfies:
  $$\psi_{t}=\psi_{0}+\alpha$$
  where, $\psi_{0}$ is the current heading angle of AUV, $\psi_{t}$ is the heading angle when AUV is arriving at *S*_1_.
Step 7: End
Step 8: Replace the current line segment with the next line segment (such as: line BC instead of line AB), which is displayed in Figure 8b,c, repeat steps 1–6;
Step 9: Taking the transition point *S*_3_ as beginning position of steering, $D_{s1}\geq 2L_{o}$, $D_{s2}\geq 4L_{o}$, in Figure 8d, one can obtain:
  (21) $$\left\{ \begin{array}{l} \bar{B^{\prime }L}=D_{s1}ctg(0.5\alpha )\\ \bar{LN}=(D_{s2}-D_{s1})/\sin\alpha \end{array}\right.$$
Step 10: If $D_{s1}\geq 4L_{o}$ and $g_{1}(\alpha )$ is nonnegative, take $D_{s2}=D_{s1}$, and replacing *S*_3_ with $B^{\prime }$:
  (22) $$g_{1}(a)=\bar{B^{\prime }L}/\cos(0.5\alpha )-R_{\min}$$
  The turning radius *R* satisfies:
  (23) $$R=D_{s1}/\sin(0.5\alpha )$$
Step 11: Else, take turning radius *R* = *R*_min_, and point *S*_3_ satisfies:
  (24) $$\left\{ \begin{array}{l} g_{2}(\alpha )=R_{\min}\sin\alpha \cos(0.5\alpha )+D_{s1}\cos\alpha \\ D_{s2}=\max(g_{2}(\alpha ),4L_{o})\\ MS_{3}=D_{s2}/\sin\alpha -R_{\min}\cos(0.5\alpha ) \end{array}\right.$$
Step 12: Design a transition point *S*_4_ as steering end position, where the heading angle of the AUV satisfies:
  (25) $$\psi_{t}=\psi_{0}+\alpha$$
  where, $\psi_{t}$ is the heading angle before the AUV arrives at *S*_3_.
Step 13: Turn to step 7.

### 3.4. Wall-Form Obstacles

Norgren presented the obstacle avoidance method called “iceberg Edge-Following” in [24], however, the wall form of obstacles in this paper is too simple, and this obstacle avoidance method doesn’t adapt to complex U-shape obstacles, or labyrinth obstacles. The next question is when is the optimal time for ending this manner and driving straight to the target for complex wall form obstacles, which hasn’t been proposed in the above paper.

**Figure 9:**
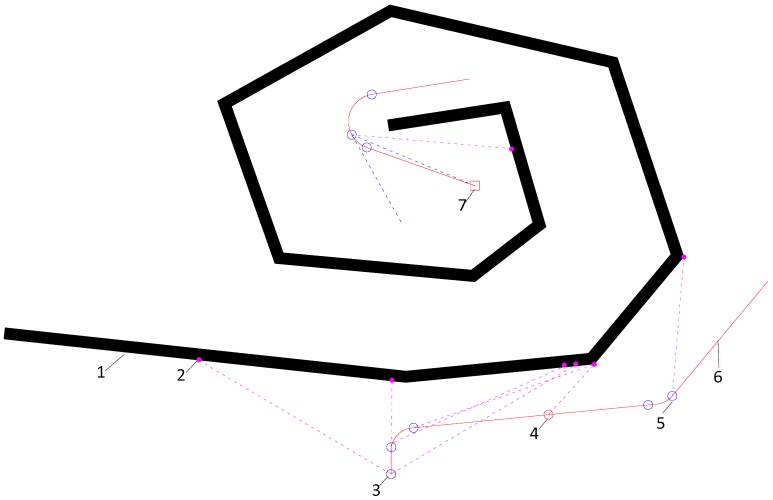
Wall-form obstacle avoidance. 1-wall form obstacle; 2-recorded obstacle point; 3-start record position; 4-supplement record position; 5-transition position; 6-obstacle trajectory; 7-target.

To solve this question, the paper presents a novel solution, which is obstacle outline memory algorithm. In Figure 9, the AUV detours around the wall-form obstacle from the right side, and once a wall-form obstacle is detected, the walking along wall manner is activated, and the memory function is started to recorded the rightmost obstacle points *S*_79_ detected by FLV at the transition position where steering is beginning or ending. Nevertheless, the wall-form obstacle outline is not described entirely by these points, so it is necessary to add other supplementary points, to ensure an obstacle point must be recorded that at least every 100 m of navigation distance. On the contrary, if the AUV detours around the wall-form obstacle from the left direction, leftmost obstacle points *S*_0_ are recorded at the transition position.

These obstacle points are kept in a storage array $D\in R^{n\times 2}$, As a demonstration in Figure 9, when the wall-form obstacles are identified and it is decided to detour around their right side, the first three obstacle points are recorded in array D in order, which are the leftmost point *S*_0_, dead ahead point *S*_39_ and rightmost point *S*_79_. Then the next obstacle point position is added into array *D* as mentioned above. This is repeated until this obstacle is passed by, and the simulation process is explained in the following section. 

The manner of walking along wall is terminated if the following two conditions are satisfied: firstly, ADT is smaller than ϑ (take ϑ = 15°); secondly, any line composed by two arbitrarily adjacent points taken from array D, which doesn’t intersect line $\vec{\mathrm{PG}}$, where, *P*(x, y) is the current position of AUV and *G*(x_t_, y_t_) is the position of target. Whenever the first condition is satisfied, an obstacle point will be recorded by the aforementioned ways at current position *P*(x, y) right now, and the second condition will be verified. Let *K*_1_ and *K*_2_ be arbitrarily two adjacent points taken from *D*, the second condition can be described by the following formulas:

(26) $$\left\{ \begin{array}{l} d_{1}=\vec{Pk_{1}}\times \vec{PG}\\ d_{2}=\vec{Pk_{2}}\times \vec{PG}\\ d_{1}\cdot d_{2}>0 \end{array}\right.$$

(27) $$\left\{ \begin{array}{l} d_{3}=\vec{k_{1}P}\times \vec{k_{1}k_{2}}\\ d_{4}=\vec{k_{1}G}\times \vec{k_{1}k_{2}}\\ d_{3}\cdot d_{4}>0 \end{array}\right.$$

where: × denotes a cross multiplication, and ⋅ denotes a scalar multiplication.

If the above Equations (26) and (27) are satisfied, which means segment $\vec{k_{1}k_{2}}$ doesn’t intersect with segment $\vec{PG}$, the second condition is satisfied, too. Then “wall-form walk” manner is terminated, and the AUV drives straight to the target.

### 3.5. Clutter Obstacles Environment

When any obstacle enters into the 80 m detection range of the AUV, and the detected obstacles are more than two, the obstacles environment is considered a cluttered obstacle environment. In this environment, the speed is slowed down in narrow channels if necessary, and it is unnecessary to limit the steering times in this obstacles scenario for three reasons, firstly, the effect of steering is decreased steeply with the speed decline; and there is not enough space for the AUV turn round freely either; finally, sometimes obstacle avoidance can’t be accomplished without the assistance of auxiliary thrusters.

There are several candidate detouring paths in dense obstacles environments, so a weight coefficient $\lambda_{c}$ is designed to evaluate these obstacle avoidance paths, and the path whose weight coefficient $\lambda_{c}$ is the largest is chosen as the optimal path, and $\lambda_{c}$ denoted as:

(28) $$\lambda_{c}=\frac{\lambda_{b}\lambda_{o}L_{e}}{\rho_{d}\sin\varphi_{d}}$$

(29) $$\rho_{d}=\left\{ \begin{array}{l} \rho_{i},\mathrm{first}\;\mathrm{type}\;\mathrm{gap}\\ 0.5(\rho_{i}+\rho_{i+1})，\mathrm{second}\;\mathrm{type}\;\mathrm{gap} \end{array}\right.$$

(30) $$\lambda_{o}=\left\{ \begin{array}{ll} 0, & D_{o}\leq 4L_{o}\\ {\left(\frac{D_{o}-4L_{o}}{{8L}_{o}}\right)}^{1/2}, & 4L_{o}<D_{o}<8L_{o}\\ 1, & D_{o}\geq 8L_{o} \end{array}\right.$$

where, $D_{o}$ is the gap width, $\varphi_{d}$ is ADT, $\rho_{i}$, $\rho_{j}$ are the distances from FLV to the right or left border points of the gap respectively, $\lambda_{o}$, $\lambda_{b}$ are the width coefficient of the gap, and type coefficient of the gap.

Considering the detouring path forming a gap to the target may be obstructed, which can’t be identified directly from current visual angle of FLS, so the type coefficient is introduced as the reliability factor. If there is a detection point *S_i_* between a gap that satisfies the condition *S_i_* = 0, which means the gap is unobstructed from the current visual of angle FLV, take *λ_b_* = 1; otherwise take *λ_b_* = 0.5. The weight function *λ_c_* is considered a comprehensive factor that includes safety, reliability and efficiency. After the detour direction is decided, a temporary waypoint needs to be designed, which is solved by the following rules:

(a) If the width of gap is smaller than 8 *L_o_*, choosing the midpoint of the gap as waypoint;
(b) If the width of gap is larger than 8 *L_o_*, choosing the point as waypoint where the ADT is the least and the distance from AUV to two border points of the gap is larger than 4 *L_o_*.

**Figure 10:**
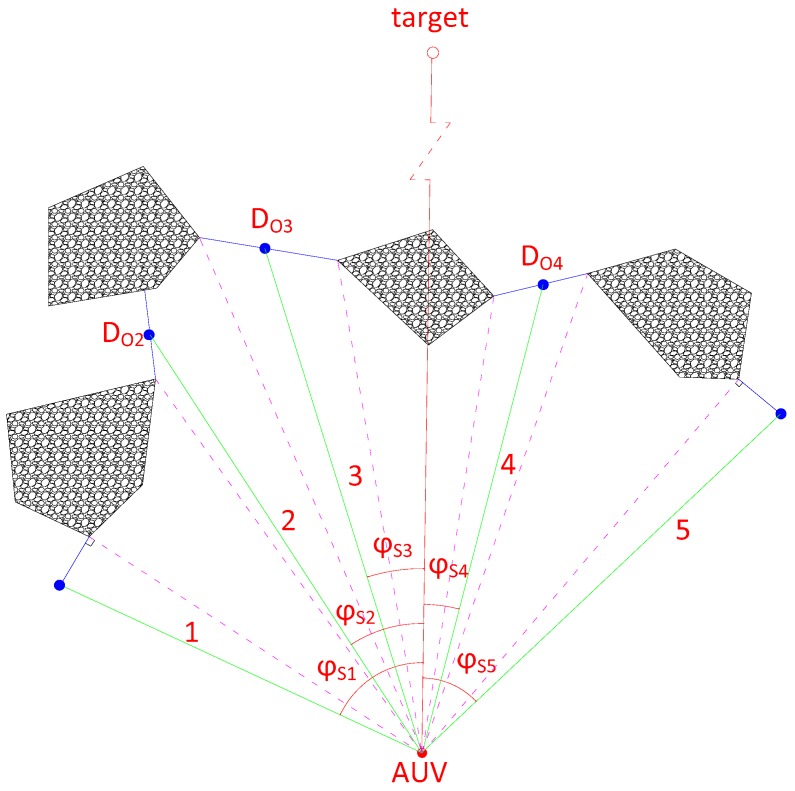
Dense obstacles environment voidance.

When the width of path less than 6 *L_o_* the AUV should navigate at low speed, In Figure 10, the AUV has five paths, and the weight function and the parameters give the following table:

From Table 1, we can get the 4th path is the optimal path and the 2nd path is the worst one.

## 4. Simulation Results and Discussion

Numerical simulations have been performed in three obstacle environments, and three different obstacle avoidance algorithms are adapted in order to verify the effectiveness of the proposed obstacle-avoidance algorithm in complex environments. and software simulation experiments are carried out in the Matlab 2015 environment. We design three obstacle scenes, and the obstacles are irregular shape, the time step takes 0.1 s. The other parameters are as follows:

*l_o_* = 4 m, *U* = 2 m/s, *R*_max_ = 30 m, *R*_min_ = 30 m, *λ*_2_ = −0.663, *λ_b_* = 2.0.

### 4.1. Simulation

**Figure 11:**
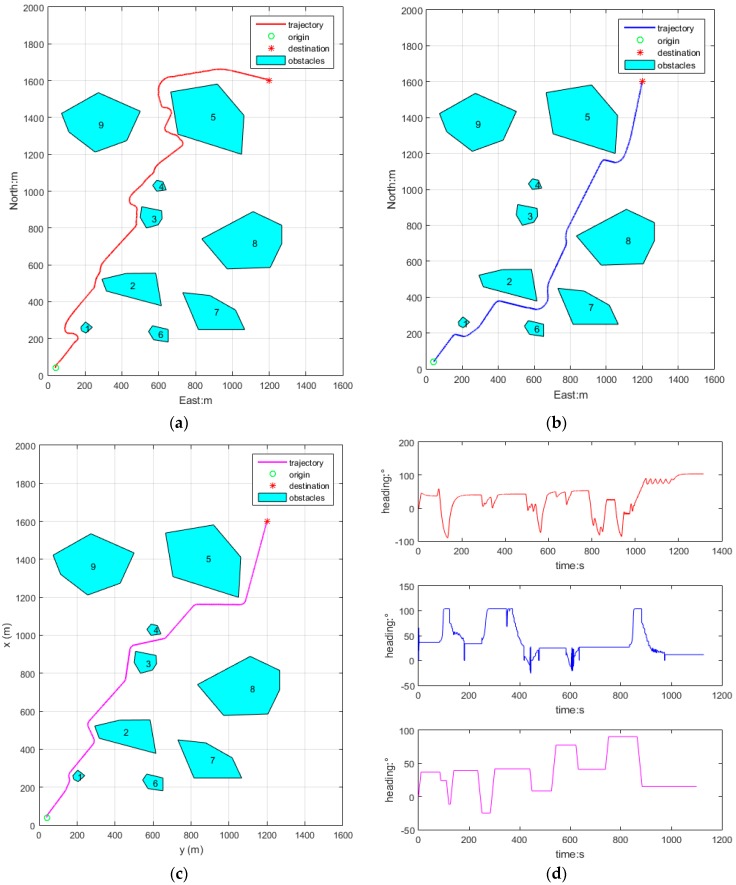
General obstacles environment obstacle avoidance algorithms. (**a**) APF; (**b**) PSO; (**c**) RRA; (**d**) heading curves.

Figure 11 depicts a AUV’s obstacle avoidance in a general obstacle environment, initial position (40, 40), heading angle is 0° (north), and initial speed is 2 m/s, the target position is (1200, 1600), initial time *t* = 0 s. As the obstacles are dispersely distributed, the AUV maintains a speed of 2 m/s in the whole process ignoring the steering effect on speed.

Figure 11a depicts the avoidance trajectory of APF, the trajectory obviously isn’t good, it has some defects in the following aspects: the path is not smooth; and the path is too long and the heading is not steady.

Figure 11b depicts the obstacle avoidance trajectory of PSO, where the trajectory is smooth, and the path length is shorter than with the first algorithm, however, the heading shows a slight shock when the heading is suddenly altered. 

Figure 11c depicts the obstacle avoidance trajectory of RRA, where the trajectory is smooth, the heading is steady and the path length is near optimal. 

Figure 11d shows the heading curves of APF, PSO and RRA, respectively, and their heading stabilities are improved in sequence, and the finishing times are 1315.4 s, 1128.7 s, 1100.1 s, respectively. Therefore, RRA is the best one of the three obstacle avoidance algorithms in every aspect.

Figure 11 depicts the AUV’s obstacle avoidance in a cluttered obstacle environment, where the initial position is (20, 20), heading angle is 0° (north), and initial speed is 2 m/s, the target position is (600, 800), initial time *t* = 0 s.

Figure 11a depicts obstacle avoidance trajectory of APF. The path is not smooth and it is too long and the heading is not steady and oscillates when navigating in a narrow passageway. 

Figure 11b depicts the obstacle avoidance trajectory of PSO, where the trajectory is not smooth like the first scene, and the path length is shorter than the APF, and the heading displays a slight shock when the heading is suddenly altered. 

Figure 11c depicts the obstacle avoidance trajectory of RRA, where the trajectory is still smooth, the heading is steady and the path length is near optimal. 

Figure 11d shows the heading curves of APF, PSO and RRA, respectively, and their heading stabilities is still improved in sequence, and the finishing times are 642.0 s, 571.1 s, 560.1 s. Therefore, RRA is the best one among the three obstacle avoidance algorithms in every aspect.

**Figure 12:**
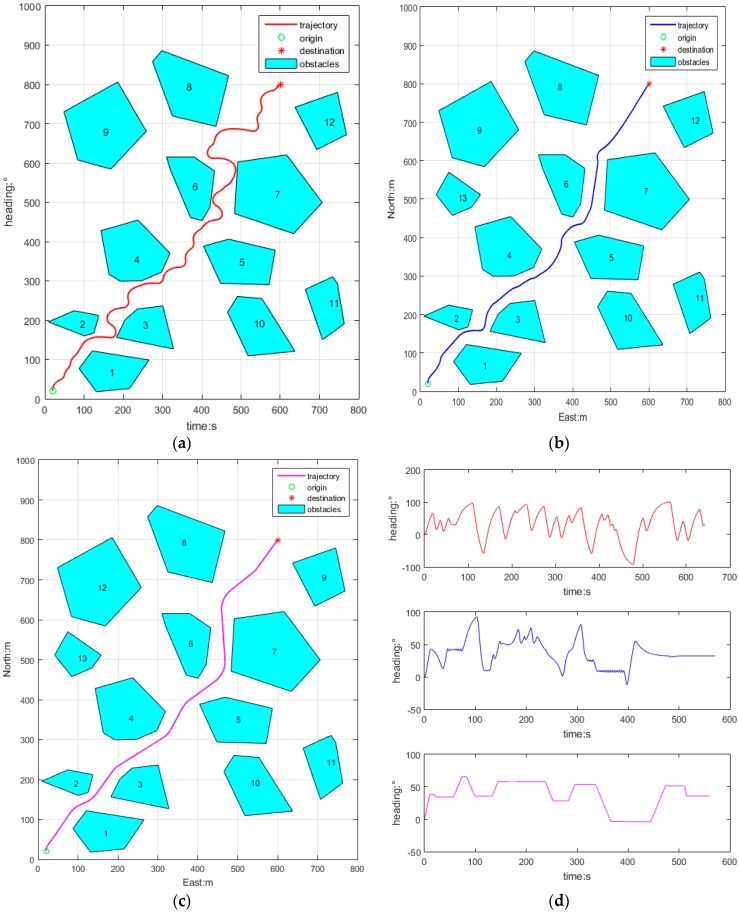
Clutter obstacles environment obstacle avoidance algorithms; (**a**) APF; (**b**) PSO; (**c**) RRA; (**d**) heading curves.

In a cluttered obstacle scene, maintaining the safety distances between the AUV and obstacles is an important factor, Figure 12 displays the smallest distances between the AUV and nine detected obstacles by three obstacle avoidance algorithms. The three columns are the smallest distances in APF, PSO and RRA, respectively. 

The smallest distance in APF is only 6.01 m, produced by an AUV detouring the 3rd obstacle. The smallest distance in PSO is 10.38 m produced in an AUV detouring the 2nd obstacle, and the smallest distance in PSO is 16.50 m produced in an AUV detouring the 7th obstacle. The distance between the AUV and obstacles is bigger than twice the total length of the AUV in the obstacle avoidance process which is safe enough, and more than four times the total length is perfect, so RRA is still the best one in keeping a safe distance.

**Figure 13:**
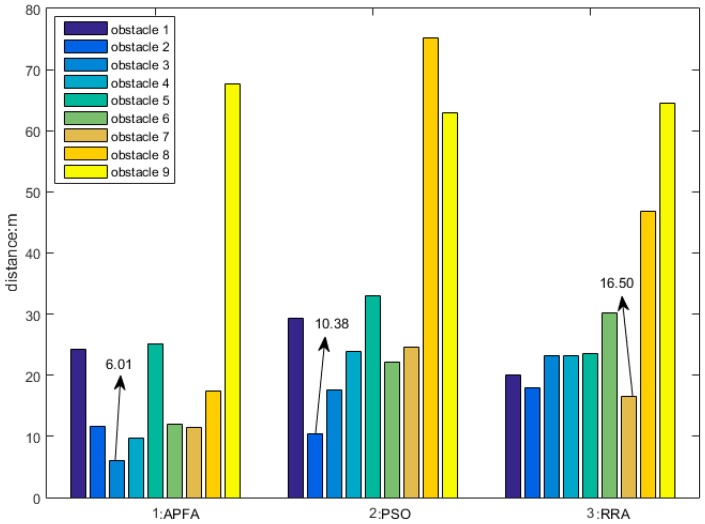
Smallest distance between AUV and obstacles.

Figure 13 displays the obstacle avoidance trajectories of the three algorithms in a trap obstacles environment, where the initial position is (700, 1050), the heading angle is 0° (north), the initial speed is 2 m/s, and the target position is (970, 1820), initial time *t* = 0 s. 

**Figure 14:**
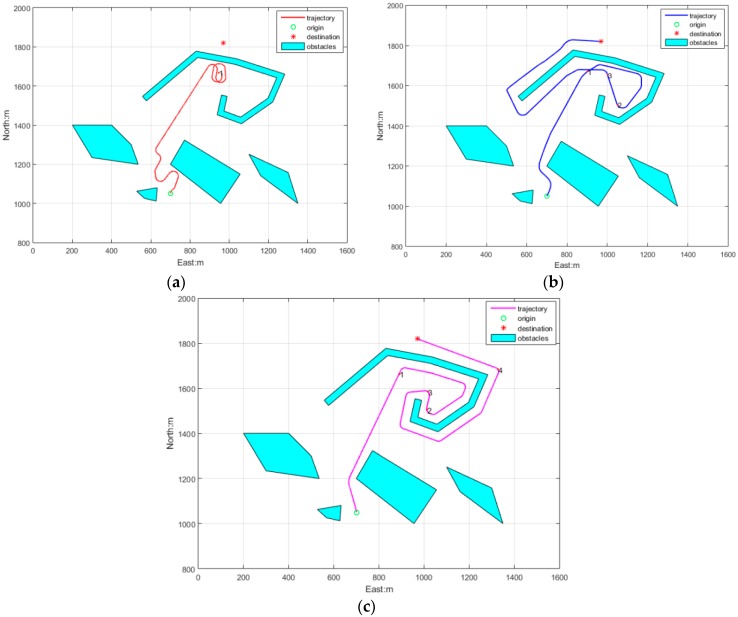
Trap obstacles environment obstacle avoidance algorithms: (**a**) APF; (**b**) PSO; (**c**) RRA.

Figure 14a depicts the APF obstacle avoidance trajectory, as the inherent defect of APF, AUV is being caught in the trap and they wander around position 1. 

Figure 14b depicts the PSO obstacle avoidance trajectory, where although the AUV arrived at the destination, it made some detours in this method. When the AUV arrived at position 1 it detected a wall-shape obstacle and start walking along the wall, and when AUV arrived at position 2 the walking along wall was ended and it went to destination directly, however when the AUV arrived at position 3 and detected a wall obstacle again, it restart walking along wall again till arriving at the destination. 

Figure 14c depicts the PSO obstacle avoidance trajectory, where the path is smooth, and the length is shorter. When the AUV arrived at position 1, the obstacle was identified as a wall-form obstacle, the walking along wall manner was activated and obstacle points were recorded frequently by the aforementioned rules. When the AUV arrived at position 2 and position 3, the heading was pointing to the destination, but the second condition was not satisfied, so it continued maintaining this manner. When the AUV arrived at position 4, the heading was pointing to the destination again, and the second condition was not satisfied, so walking along wall manner was terminated and the AUV drived to the target directly.

### 4.2. Discussion

Simulations have been carried out in three different unknown obstacle scenarios. The obstacle avoidance trajectory by APF is worse, the path is not smooth and heading is not steady, and paths are the longest in all three obstacle scenes using this algorithm, moreover, the AUV can’t get to the destination in the third scenario with this algorithm. The obstacle avoidance trajectory by PSO is good in general, however, if a U-shape obstacle is more complicated, for example, in Figure 14b the left part of wall-form obstacle was as complicated as its right side, AUV could get trapped inside a wall-form obstacle by this algorithm. It is apt to become trapped in complicated U-shape obstacle environments which it cannot solve by itself. The RRA is superior in the following aspects: it can realize the goals in all three different obstacle scenes with the shortest time, respectively, planning smooth trajectories and the shortest voyages, meanwhile, it makes the AUV keep a steady heading, keeping better safe distances away from obstacles. Moreover it solves the defect of being apt to getting into traps in U-shape obstacle environments that affects APF.

## 5. Conclusions and Future Work

In this paper, a LPAA had been proposed to change an obstacle’s irregular visual surface into a convex polygon, and a real-time reaction obstacle avoidance algorithm for AUVs is presented. The algorithm adapts to complex and cluttered unknown environments, and generates a smooth and shorter path, while guaranteeing appropriate safe distances. The simulation experiments illustrate that this algorithm is flexible in cluttered unknown environments, and in particular, it is able to solve the problem of AUVs becoming trapped by U-shape obstacles existing in other obstacle avoidance algorithms. The next stage of this work is to apply this algorithm in pool experiments, and we will extend this algorithm to more complicated ocean environments, where time variable ocean currents and dynamic objects exist.

## Figures and Tables

**Table 1:** The weight factors of path in clutter environment.

	*λ_o_*	*D_o_* (*L_o_*)	*Φ_d_* (*°*)	*P_d_*	*λ_b_*	*λ_c_*
Path1	1	∞	66	38.1	1	4.31
Path2	0.84	9.6	34	47.5	0.5	2.36
Path3	1	14.2	18	51.9	1	9.36
Path4	0.81	9.2	14	46.5	1	10.76
Path5	1	∞	43	47.4	1	4.64
